# Variations in gender identity and sexual orientation of university students

**DOI:** 10.1093/sexmed/qfad057

**Published:** 2023-11-11

**Authors:** Tomoko Yoshida, Keiko Matsubara, Hiroko Ogata-Kawata, Mami Miyado, Keisuke Ishiwata, Kazuhiko Nakabayashi, Kenichiro Hata, Ikuko Kageyama, Satoshi Tamaoka, Yukiko Shimada, Maki Fukami, Shoko Sasaki

**Affiliations:** Department of Molecular Endocrinology, National Research Institute for Child Health and Development, Tokyo, 157-8535, Japan; Department of Molecular Endocrinology, National Research Institute for Child Health and Development, Tokyo, 157-8535, Japan; Department of Maternal-Fetal Biology, National Research Institute for Child Health and Development, Tokyo, 157-8535, Japan; Department of Molecular Endocrinology, National Research Institute for Child Health and Development, Tokyo, 157-8535, Japan; Department of Maternal-Fetal Biology, National Research Institute for Child Health and Development, Tokyo, 157-8535, Japan; Department of Maternal-Fetal Biology, National Research Institute for Child Health and Development, Tokyo, 157-8535, Japan; Department of Maternal-Fetal Biology, National Research Institute for Child Health and Development, Tokyo, 157-8535, Japan; Department of Molecular Endocrinology, National Research Institute for Child Health and Development, Tokyo, 157-8535, Japan; Department of Molecular Endocrinology, National Research Institute for Child Health and Development, Tokyo, 157-8535, Japan; Department of Child Studies, Faculty of Human Development, Kokugakuin University, Kanagawa, 225-0003, Japan; Department of Molecular Endocrinology, National Research Institute for Child Health and Development, Tokyo, 157-8535, Japan; Graduate School of Arts and Letters, Meiji University, Tokyo ,101-0064, Japan

**Keywords:** gene, homosexuality, SKAT-O, SNP, transgender, young adult

## Abstract

**Background:**

Previous studies have shown that a small percentage of people in the general population have atypical gender identity and/or sexual orientation.

**Aim:**

This study aimed to explore variations in gender identity and sexual orientation in university students and determine genetic factors associated with these variations.

**Methods:**

Deviations from complete gender congruence and exclusive heterosexual orientation in 736 Japanese university students were quantitatively assessed with self-assessment questionnaires. Next, we conducted genetic tests for 80 participants who showed relatively low gender identity scores and/or atypical sexual orientation. These genetic tests consisted of repeat number analysis of the androgen receptor gene (*AR*) and a SKAT-O: an optimal unified sequence kernel association test, which is an exome-based rare variant association study. The results of the genetic tests were compared with the Japanese reference data and the results of our 637 control samples.

**Outcomes:**

We calculated the gender identity and sexual orientation scores of all participants and analyzed the molecular data of 80 selected participants.

**Results:**

The gender identity scores of 736 participants were broadly distributed: only ~15% of natal males and ~5% of natal females had the maximum score that corresponds to complete gender congruence. The sexual orientation scores also varied: ~80% of natal males and ~60% of natal females showed exclusive heterosexual orientation. We found no association between gender characteristics and *AR* repeat numbers. The SKAT-O showed that rare damaging variants of *TDRP* and 3 other genes were more common in the 80 participants than in the control group.

**Clinical Implications:**

Our data support the view that gender is a phenotypic continuum rather than a binary trait.

**Strength and Limitations:**

This study quantitatively assessed the gender characteristics of a large cohort of university students. Moreover, we conducted systematic screening for genetic factors associated with gender variations. The weaknesses of the study were the limited analytic power of the questionnaires, the relatively small sample for molecular analyses, and incomplete clinical information and relatively advanced ages of the control group.

**Conclusion:**

This study revealed significant variations in gender identity and sexual orientation in university students, which may be partly associated with variants in *TDRP* or other genes.

## Introduction

Gender identity and sexual orientation are fundamental components of sexual identity.^1^ Gender identity is defined as the sameness, unity, and persistence of one’s individuality as male, female, or ambivalent.^2^ Types of gender identity include cisgender, transgender, nonbinary, and gender fluid.^3^ Sexual orientation indicates a person’s physical, emotional, and romantic attachments in relation to gender.^4^ Sexual orientation includes heterosexual, homosexual, bisexual, and asexual.^4^ Gender identity and sexual orientation are independent traits. However, considering that natal male adolescents and adults with gender dysphoria on early onset are almost always androphilic and natal females are almost always gynephilic,^5^ gender identity and sexual orientation are likely to be partially related features. Previous studies have highlighted the presence of people with atypical gender identity or sexual orientation.^6^–8 For example, the proportion of people identified as transgender or nonbinary gender in Brazil was 1.9%.^9^ Yet, only a few studies have addressed the variations in gender characteristics of people in the general population.^10^

Gender identity and sexual orientation are known to be determined by genetic, hormonal, and neuroanatomic factors, as well as by sociocultural and experiential factors.^11^ Twin studies suggested that genetic factors play an important role in the development of gender incongruence and same-gender sexual orientation^12^ and that there may be genetic overlap between these phenotypes.^13–15^ In particular, relatively long CAG repeats in the androgen receptor gene (*AR*) on Xq12 have been linked to gender incongruence by several researchers.^16^^,^^17^ The expansion of CAG repeats is assumed to reduce the binding activity of the AR protein to cofactors, thereby resulting in less effective testosterone signaling.^18^ Moreover, genome-wide association studies identified various genomic loci correlated with same-gender sexual orientation.^19^^,^^20^

In the present study, we addressed 2 questions: (1) Are there any variations in gender identity and sexual orientation in the general population? (2) Are gender identity and sexual orientation driven by the same genetic factors? To this end, we conducted a quantitative assessment of gender identity and sexual orientation for 736 university students. Subsequently, we performed genetic tests for participants with relatively low gender identity scores and/or atypical sexual orientation to determine genetic factors associated with these variations.

## Methods

### Participants

The participants of this study were recruited from 3 universities in Japan. We announced this project to students after lectures. These lectures were randomly selected from the university programs. We invited all students >20 years of age, and they freely decided whether to participate. Only students who provided written informed consent were included in this study. As a result, 736 students voluntarily participated. They anonymously filled out paper-based questionnaires and provided saliva samples for genetic analysis. Each participant received 1000 yen for his or her efforts in sample preparation. The participants were classified according to assigned sex at birth into the male group (n = 313) and female group (n = 423) (hereafter, males and females). To assess the results of genetic analyses, we consulted Japanese reference data and our in-house data from healthy Japanese individuals.

This study was approved by the Institutional Review Board Committee at the National Center for Child and Development and performed after obtaining informed consent from all participants. The questionnaires and saliva specimens were anonymized immediately after sampling.

### Assessment of gender identity and sexual orientation

The gender identity and sexual orientation of the 736 participants were assessed with paper-based self-assessment questionnaires.^21^^,^^22^ These were developed to quantify the deviation from complete gender congruence and exclusive heterosexual orientation.^21^ We used the original version written in Japanese. The validity of these questionnaires was confirmed in a previous study on 316 university students and 175 transgender people.^21^

**Figure 1:**
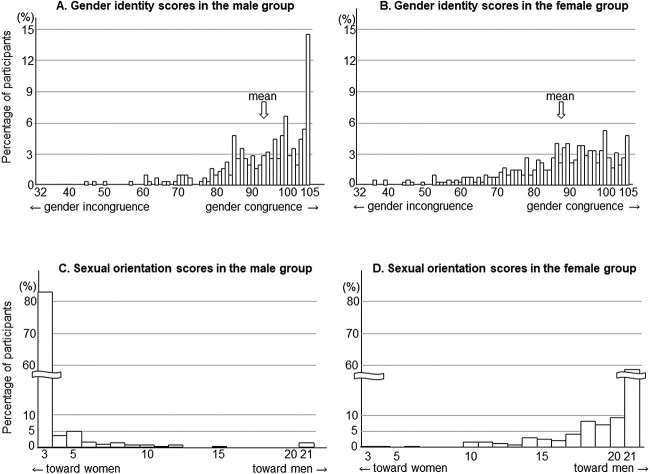
The results of self-assessment questionnaires. Distributions include gender identity scores (A, B) and sexual orientation scores (C, D). Participants were classified into male (A, C) and female (B, D) groups according to the assigned sex at birth. Individuals whose self-recognized gender was nonbinary or opposite to the assigned sex were excluded from this figure. A higher gender identity score indicates gender congruence. Lower and higher sexual orientation scores depict sexual orientation toward women and men, respectively.

First, we analyzed the gender identity of each participant—specifically, whether one’s self-recognized gender was man, woman, or nonbinary. Then, the participants were asked to provide answers to 15 items (for the English version of this questionnaire, see Ikuta et al^6^ and Hisasue et al^22^). These items were the same for men, women, and nonbinary individuals, with the exception of the wording of his or her own gender (“man” and “woman”). The answers to each item were selected from “very true” (score 7) to “not true at all” (score 1). The sum of the scores to the 15 items was defined as the gender identity score, ranging from 15 (obvious gender incongruence) to 105 (complete gender congruence). We created distribution histograms of the scores of the male and female groups (Figure 1). Individuals whose self-recognized gender was nonbinary or opposite to the assigned sex were excluded from these histograms.

Next, we assessed the sexual orientation of each participant. We used a questionnaire consisting of 6 items: sexual attraction, sexual behavior, sexual fantasies, emotional preference, social preference, and affection. Five of the 6 items were adopted from the Sexual Orientation Grid created by Klein et al,^23^ which has been widely utilized in several studies.^24^^,^^25^ The remaining item (affection) was newly included in this questionnaire. Although the original grid by Klein et al assesses sexual orientation in the past and present and as an ideal, we analyzed sexual orientation only at the present. Some wordings were modified from the Klein Sexual Orientation Grid to be more appropriate for the transgender and nonbinary participants. Specifically, we replaced the words “the same sex” and “the other sex” with “man” and “women.” The answer options consisted of sexual orientation toward “women only” (score 1), “women mostly” (score 2), “women somewhat more than men” (score 3), “both sexes equally” (score 4), “men somewhat more than women” (score 5), “men mostly” (score 6), and “men only” (score 7), as well as “nonbinary,” “not certain,” and “I have no experience.” After initial data analysis, we decided to focus on 3 of the 6 items (sexual attraction, sexual fantasies, and emotional preference) for principal component analysis because these items showed high factor loadings (0.96-0.97). Social preference and affection were excluded from the principal component analysis because of low factor loadings (0.31 and 0.65, respectively), and sexual behavior was excluded because >30% of the participants chose “not certain” or “I have no experience” for this item. Consequently, the sexual orientation score of each participant ranged from 3 (sexual orientation exclusively toward women) to 21 (exclusively toward men). We created distribution histograms of the scores of the male and female groups (Figure 1). Those who answered that they had nonbinary or uncertain sexual orientation or lacked sexual experience were excluded from these histograms.

### Identification of individuals with low gender identity scores and/or atypical sexual orientation

We aimed to examine whether gender variations in our participants are associated with sequence variants in specific genes. To this end, we first selected participants who showed relatively large deviations from complete gender congruence: (1) those whose self-recognized gender was opposite to the assigned sex, (2) those who answered that his or her self-recognized gender is nonbinary, and (3) those whose gender identity scores were below the fifth percentile of all participants of this cohort with the same assigned sex.

Next, we searched for participants with a tendency toward same-sex sexual orientation. From the male group, we chose (1) participants whose scores were 5 to 8 or “nonbinary” for at least 1 of the 3 items and (2) participants who selected a score of 4 for ≥1 of the 3 items and did not select a score of 1 or 2 for the remaining items. From the female group, we selected (1) participants whose scores were 1 to 3, 8, or “nonbinary” for at least 1 of the 3 items and (2) participants who selected a score of 4 for ≥1 of the 3 items and did not select a score of 6 or 7 for the remaining items.

### Molecular analysis for individuals with low gender identity scores and/or atypical sexual orientation

Saliva samples were collected between January 2019 and February 2020. Genomic DNA was extracted with the Oragene-Discover kit (DNAgenotek). First, we analyzed the CAG repeat number polymorphism in *AR* with an ABI Prism 3130 Genetic Analyzer (Applied Biosystems) and GeneMapper 3.7 software (Applied Biosystems). The methods were reported previously.^26^ For female participants, we calculated the mean number of *AR* alleles on the two X chromosomes. The results of the participants were compared with the reference data of Japanese individuals.^26^

Second, we conducted an optimal unified sequence kernel association test (SKAT-O)^27^ to detect rare variants that were more commonly present in the selected participants than in the controls, for which we used in-house whole exome data. These data were obtained from healthy Japanese people (329 females and 308 males) who had a heterosexual partner and at least 1 child. The controls were mostly in their twenties, thirties, or forties. We selected 205 putative target genes that have been associated with gender incongruence, nonheterosexual orientation, disorders of sex development, sex hormone biosynthesis, the hypothalamus-pituitary-gonadal axis, regulation of puberty, sex-specific expression in the brain, or the 2D:4D digit ratio.^16^^,^^19^^,^^20^^,^^28^–52 The 2D:4D digit ratio was reported as a marker for prenatal androgen exposure.^50^ We designed a custom target enrichment panel (HaloPlex HS; Agilent Technologies) with SureDesign (Agilent Technologies) to construct DNA libraries. Since 29 of the 205 genes showed low coverage rates, only the remaining 176 genes were examined in this study (Table S1). Four Y chromosomal genes were analyzed only in males. DNA libraries were sequenced on a NextSeq sequencer (Illumina). VCF files were created with the SureCall system (Agilent Technologies) and analyzed with the SNP & Variation Suite (version 8.4.1; Golden Helix). We focused on putative protein-altering variants that were assessed as deleterious or unknown significance by ≥1 of 6 in silico programs (version 3.0, http://database.liulab.science/dbNSFP/). Common polymorphisms whose allele frequency in the ToMMo database^53^ (version 8.3KJPN; https://www.megabank.tohoku.ac.jp/) is >1.0% were excluded from further analysis. The results were adjusted through Bonferroni correction, and adjusted *P* values <.05 were considered significant.

### Statistical analysis

Statistical differences in the median scores between the male and female groups were assessed by the nonparametric Mann-Whitney *U* test. The frequencies of sequence variants between the participant and control groups were compared with the Fisher exact test. Both analyses were performed with R (version 3.3.2).

## Results

### Assessment of gender identity and sexual orientation

In 726 of 736 participants (98.6%), self-recognized gender was consistent with sex assigned at birth, whereas 2 of 313 males and 7 of 423 females were assessed as having nonbinary gender (0.6% vs 1.7%, *P* = .31). One from the female group had already been recognized as a transgender person. The results of the 726 individuals are shown in Figure 1 (upper panel). There were large interindividual variations in the male and female groups. In the male group, gender identity scores ranged from 45 to 105. The median score was 96 of 105, and only 46 participants (14.7%) showed the maximum score of 105, which corresponds to complete gender congruence. In the female group, the scores ranged from 32 to 105. The median score was 89 of 105, and just 20 participants (4.7%) had the maximum score. The median scores, as well as the rate of people with the maximum score, were higher in males than in females (*P* = 1.8 × 10^–10^ and *P* = 3.5 × 10^–6^, respectively).

Sexual orientation scores were obtained from 313 males and 423 females. Of these, 13 males and 90 females were excluded from data analyses because they had nonbinary gender or uncertain sexual orientation or lacked sexual experience at least for 1 of the 3 items. The results of 633 individuals (300 males and 333 females) are shown in Figure 1 (lower panel). Variations in sexual orientation scores were relatively small as compared with those in gender identity scores. Specifically, 250 males and 195 females had exclusive heterosexual orientations (83.3% vs 58.6%, *P* = 6.6 × 10^–12^). An exclusive same-sex sexual orientation was observed in 4 males and 1 female (1.3% vs 0.3%, *P* = .20).

### Identification of individuals with low gender identity scores and/or atypical sexual orientation

We selected 83 participants who had relatively low gender identity scores and/or atypical sexual orientation. Of these, 29 and 35 had low gender identity scores and atypical sexual orientation, respectively, while 19 showed both features. Among them, 80 (27 males and 53 females) were subjected to molecular analyses; the remaining 3 were not examined because the amounts of DNA samples were insufficient for systematic sequencing.

### Molecular analysis for individuals with low gender identity scores and/or atypical sexual orientation

We examined CAG repeat numbers in *AR* in the 80 selected participants. The mean ± SD of the repeat number in the male and female groups was 19.2 ± 2.55 and 19.5 ± 2.23, respectively (Figure S1). These numbers were not larger than the reference data of Japanese individuals (males, 23.7 ± 0.46; females, 23.2 ± 0.23).^26^ Moreover, none of the 80 participants carried extremely long repeats (≥26).

**Figure 2:**
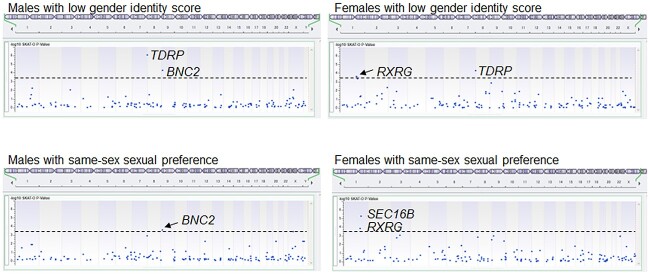
Manhattan plots of the optimal unified sequence kernel association test. Results show 80 participants with relatively low gender identity scores and/or same-sex sexual orientation. The broken lines depict the cutoff values. Four genes showed possible association with the gender variations.

Sequence analysis of 176 genes identified 1849 rare damaging variants in the participants or controls. We excluded variants in *CYP21A2* because the mapping accuracy of this gene was low. SKAT-O identified 4 genes whose variants were more frequently detected in the participants with high gender variation scores than in the controls (Table 1, Figure 2). In the male group, *BNC2* was associated with low gender identity scores and with same-sex sexual orientation, while *TDRP* was associated only with low gender identity scores. In the female group, *RXRG* was associated with both gender variations, while *TDRP* and *SEC16B* were associated with only low gender identity scores and same-sex sexual orientation, respectively.

**Table 1:** Genes associated with gender variations in our cohort.

	**Assigned sex at birth**			
	**Male**		**Female**	
Gender variation	Low gender identity score	Same-sex sexual orientation	Low gender identity score	Same-sex sexual orientation
No. of participants				
Tested	18	11	28	40
Control	308		329	
Associated genes^a^	*TDRP* (1.5 × 10^-4^)	*BNC2* (.042)	*TDRP* (8.3 × 10^-4^)	*SEC16B* (9.3 × 10^-4^)
	*BNC2* (.0085)		*RXRG* (.041)	*RXRG* (.024)

a Bonferroni-adjusted *P* value in parentheses.

## Discussion

Self-assessment questionnaires revealed significant variations in gender identity and sexual orientation in 736 university students. The gender identity scores of the participants were broadly distributed without yielding a clear cutoff value between gender congruence and incongruence. Only ~15% of the male group and ~5% of the female group had the maximum score of 105, indicating that complete gender congruence is relatively rare in the general population. Similarly, sexual orientation scores varied among participants, and 16.7% of the male group and 41.1% of the female group showed the tendency of same-gender sexual orientation. These results are almost comparable to those of the previous study in Italy.^10^ Our data support the view that gender identity and sexual orientation are phenotypic continuums rather than binary traits.^54^ In addition, the results of this study suggest that natal females tend to have larger variations in gender identity and sexual orientation than natal males.

Next, we selected individuals with relatively low gender identity scores and/or atypical sexual orientation. A total of 83 were identified according to our selection criteria. Of these, 19 had variations in both gender identity and sexual orientation, while the remaining 64 had variations only in 1 of the 2 features. This indicates that gender identity and sexual orientation are principally diverse traits but partly driven by some common factors.

We performed molecular analyses for 80 participants with relatively low gender identity scores and/or atypical sexual orientation. First, we analyzed a repeat number polymorphism in *AR*, which has frequently been linked to gender incongruence.^16^^,^^17^ However, our results argue against a significant association between this polymorphism and low gender identity scores or same-sex sexual orientation. Second, we performed SKAT-O for several other genes. As a result, we identified 4 genes whose variants were more frequently detected in participants with gender variations than in controls. Of these, *TDRP* was associated with low gender identity scores in both sexes. *TDRP* is a widely expressed gene^55^ whose somatic variant has been identified in a case of gender dysphoria.^46^ Thus, *TDRP* variants may affect gender identity in the general population to some extent. In addition, *BNC2* variants were associated with low gender identity scores and same-sex sexual orientation in males, while *RXRG* variants were linked to these features in females. These results imply that some genetic variants can affect gender identity and sexual orientation. *BNC2* is known to be involved in male genital development,^34^ and *RXRG* is associated with the age of menarche.^41^ The association between these variants and gender phenotypes needs to be examined in future studies.

### Strengths and limitations

To our knowledge, this is the first report of quantitative assessments of gender variations in a large cohort of the general population. In addition, we assessed genetic factors associated with gender variations.

This study has several limitations. First, our questionnaires were not sufficient to classify detailed gender characteristics such as agender and bigender. Moreover, because of the cross-sectional nature of this study, we could not address gender fluidity.^3^ In addition, some items of sexual orientation could not be subjected to the principal component analysis because of low factor loadings and the lack of sexual experience in many participants. This issue likely reduced the analytic power of the questionnaires. Second, we did not measure hormone values in the blood or saliva samples, although circulating sex hormones are known to contribute to gender variations.^11^ Third, the sample number for molecular analysis was small. Hence, some gender-associated genes may have been overlooked. Last, information on the control group was limited. If the control group included several transgender men/women or people with same-gender sexual orientation, this might have lowered the sensitivity of the SKAT-O. In addition, the ages of most controls were higher than those of the participants. In this regard, we did not analyze our participants with complete gender congruence and exclusive heterosexual orientation as the control group because their number was small, particularly in the female group. These limitations may affect the generalizability and comprehensive understanding of our findings. The results of this study need to be validated in future large-scale studies.

## Conclusion

This study uncovered significant variations in gender identity and sexual orientation in university students. The results indicate that gender is a phenotypic continuum rather than a binary trait. The diversity of gender in our participants cannot be ascribed to the CAG repeat polymorphism in *AR* but may partly be associated with rare variants in *TDRP* or other genes. Our data merit further validation.

## Author contributions

T.Y.: conceptualization, data curation, investigation, methodology, software, visualization, writing (original draft and editing). K.M.: data curation, investigation, writing (review and editing). H.O.-K.: investigation, software, writing (review). M.M.: investigation, writing (review and editing). K.I.: investigation, writing (review). K.N.: investigation, resources, writing (review). K.H.: investigation, resources, writing (review). I.K.: investigation, writing (review). S.T.: investigation, writing (review). Y.S.: investigation, writing (review and editing), supervision. M.F.: conceptualization, funding acquisition, investigation, project administration, resources, writing (original draft and editing), supervision. S.S: conceptualization, data curation, investigation, methodology, resources, writing (review and editing), supervision.

## Funding

This study was supported by grants from the Canon Foundation, the Japan Endocrine Society, National Center for Child Health and Development (2022A-1), Takeda Science Foundation, and the Mitsubishi Foundation.

## Conflicts of interest

None declared.

## Data availability

All datasets generated during and/or analyzed during the current study are available from the corresponding author on reasonable request.

## Supplementary Material

Figure_S1_qfad057

Table_S1_qfad057
