# Supplementary material for: Variations in gender identity and sexual orientation of university students
Source: Sex Med. 2023 Nov 11;11(5):qfad057. doi: 10.1093/sexmed/qfad057 (PMC10642543; doi:10.1093/sexmed/qfad057)
Supplement: Figure_S1_qfad057 [file figure_s1_qfad057.pdf]

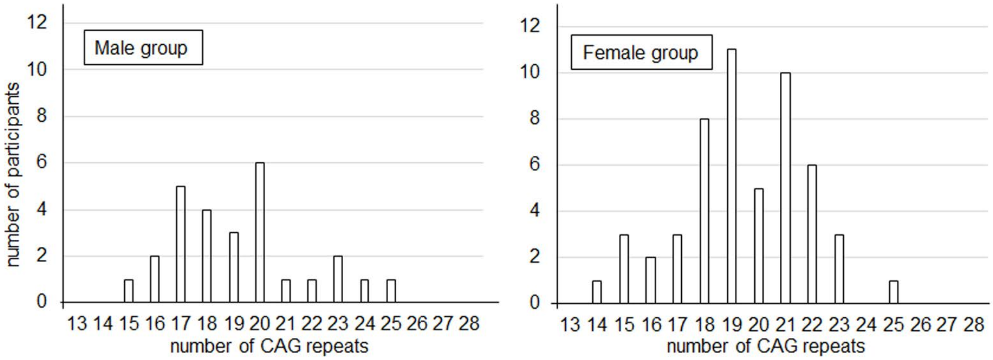

Figure S1. The number of CAG repeats of *AR* in 80 participants with relatively large gender variations. The mean ± SD of the male and female groups were 19.2 ± 2.55 and 19.5 ± 2.23, respectively.
