# Supplementary material for: Variations in gender identity and sexual orientation of university students
Source: Sex Med. 2023 Nov 11;11(5):qfad057. doi: 10.1093/sexmed/qfad057 (PMC10642543; doi:10.1093/sexmed/qfad057)
Supplement: Table_S1_qfad057 [file table_s1_qfad057.docx]

| Table S1. Genes analyzed in this study | | | | | | | | |
| --- | --- | --- | --- | --- | --- | --- | --- | --- |
| Conditions associated with the gene | Gender incongruence | Non-heterosexual orientation | Disorders of sex development | Sex hormone　biosynthesis | Hypothalamus-pituitary-gonadal axis | Regulation of pubertal onset | Sex-specific expression in the brain | 2D:4D digit ratio^a^ |
| Gene names | *COMT* | *NKAIN3* | *AKR1C2* | *AKR1C3* | *ANOS1* | *BCDIN3D* | *BRS3* | *EFNA1* |
|  | *CYP17A1* | *SLITRK1* | *AKR1C4* | *CYP11B1* | *FGF17* | *BDNF* | *CARTPT* | *FLI1* |
|  | *FBXO38* | *SLITRK5* | *AMH* | *CYP21A2* | *FGF8* | *CADM1* | *CCKAR* | *GLI3* |
|  | *HSD17B6* | *SLITRK6* | *AMHR2* | *HSD11B1* | *FGFR1* | *CENPW* | *CHODL* | *GLIS1* |
|  | *KANSL1* | *TSHR* | *ARX* | *HSD11B2* | *FLRT3* | *DLK1* | *DDX3Y*^b^ | *HOXD11* |
|  | *NR0B1* | *VIPR2* | *ATF3* | *HSD17B2* | *FSHB* | *FRS3* | *DGKK* | *HOXD12* |
|  | *RYR3* |  | *ATRX* | *HSD3B2* | *GATA4* | *GNRHR* | *ECEL1* | *LDAH* |
|  | *SMOC2* |  | *BMP15* | *PAPSS2* | *GLI2* | *GPRC5B* | *GABRG1* | *LIN28B* |
|  | *SOX9* |  | *BMP4* | *SRD5A1* | *GLI3* | *INHBA* | *GLRA3* | *OLA1* |
|  | *SRD5A2* |  | *BMP7* | *SULT2A1* | *GNRH1* | *KISS1* | *IRS4* | *SALL1* |
|  | *SRY* |  | *BNC2* |  | *GNRHR* | *KISS1R* | *KDM5C* | *SALL3* |
|  | *STS* |  | *CBX2* |  | *HESX1* | *LEKR1* | *KDM5D*^b^ | *SMOC1* |
|  | *SULT2A1* |  | *CYB5A* |  | *HS6ST1* | *LGR4* | *KDM6A* | *TOX3* |
|  | *TDRP* |  | *CYP17A1* |  | *IL17RD* | *LIN28B* | *NNAT* |  |
|  |  |  | *CYP11A1* |  | *KISS1* | *MAF* | *PAK3* |  |
|  |  |  | *DGKK* |  | *KISS1R* | *NCOA7* | *RPS4Y1*^b^ |  |
|  |  |  | *DHCR7* |  | *LEP* | *NEGR1* | *SYTL4* |  |
|  |  |  | *DHH* |  | *LEPR* | *PCSK1* |  |  |
|  |  |  | *DMRT1* |  | *LHB* | *PCSK2* |  |  |
|  |  |  | *EMX2* |  | *LHX3* | *PDYN* |  |  |
|  |  |  | *FGF10* |  | *LHX4* | *PTTG1* |  |  |
|  |  |  | *FGF9* |  | *MAGEL2* | *RXRG* |  |  |
|  |  |  | *FGFR2* |  | *MKRN3* | *SEC16B* |  |  |
|  |  |  | *FKBP4* |  | *NR0B1* | *SIX6* |  |  |
|  |  |  | *FOXL2* |  | *NSMF* | *SLC22A2* |  |  |
|  |  |  | *GATA4* |  | *OTX2* | *STARD4* |  |  |
|  |  |  | *GSTM1* |  | *POU1F1* | *TAC3* |  |  |
|  |  |  | *GSTT1* |  | *PROK2* | *TAC3R* |  |  |
|  |  |  | *HHAT* |  | *PROKR2* | *TENM2* |  |  |
|  |  |  | *HOXA13* |  | *PROP1* | *THRB* |  |  |
|  |  |  | *HOXA4* |  | *SEMA3A* | *TMEM18* |  |  |
|  |  |  | *HOXB6* |  | *SOX2* | *USF2* |  |  |
|  |  |  | *HSD17B3* |  | *SPRY4* | *VDR* |  |  |
|  |  |  | *HSD3B2* |  | *TAC3* |  |  |  |
|  |  |  | *INSL3* |  | *TACR3* |  |  |  |
|  |  |  | *LHB* |  | *WDR11* |  |  |  |
|  |  |  | *LHCGR* |  |  |  |  |  |
|  |  |  | *MAMLD1* |  |  |  |  |  |
|  |  |  | *MAP3K1* |  |  |  |  |  |
|  |  |  | *MID1* |  |  |  |  |  |
|  |  |  | *NR0B1* |  |  |  |  |  |
|  |  |  | *NR2F2* |  |  |  |  |  |
|  |  |  | *NR3C1* |  |  |  |  |  |
|  |  |  | *NR5A1* |  |  |  |  |  |
|  |  |  | *NUP107* |  |  |  |  |  |
|  |  |  | *POR* |  |  |  |  |  |
|  |  |  | *RXFP2* |  |  |  |  |  |
|  |  |  | *SOX10* |  |  |  |  |  |
|  |  |  | *SOX9* |  |  |  |  |  |
|  |  |  | *SRD5A2* |  |  |  |  |  |
|  |  |  | *SRY*^b^ |  |  |  |  |  |
|  |  |  | *STAR* |  |  |  |  |  |
|  |  |  | *TSPYL1* |  |  |  |  |  |
|  |  |  | *WNT4* |  |  |  |  |  |
|  |  |  | *WT1* |  |  |  |  |  |
|  |  |  | *WTAP* |  |  |  |  |  |
|  |  |  | *WWOX* |  |  |  |  |  |
|  |  |  | *ZFPM2* |  |  |  |  |  |
|  |  |  | *ZNRF3* |  |  |  |  |  |
| References | 16, 30, 37, 46, 47, 49, 52 | 19, 20, 28 | 29, 34, 39, 44, 48 | 42, 43 | 31, 32, 38, 45 | 33, 41 | 35, 40, 51 | 36, 50 |
| Some genes are classified into multiple categories. | | | | | | | | |
| ^a^2D:4D digit ratio was reported as a marker for prenatal androgen exposure. | | | | | | | | |
| ^b^These Y chromosomal genes were analyzed only in male individuals. | | | | | | | | |
